# Fat, Sugar, and Bone Health: A Complex Relationship

**DOI:** 10.3390/nu9050506

**Published:** 2017-05-17

**Authors:** Li Tian, Xijie Yu

**Affiliations:** Laboratory of Endocrinology and Metabolism, Department of Endocrinology, State Key Laboratory of Biotherapy and cancer center, West China Hospital, Sichuan University, Chengdu 610041, Sichuan, China; tianzhuangzhuang924@126.com

**Keywords:** high-fat diet, high fructose, glucose, sucrose, bone microstructure, bone metabolism

## Abstract

With people aging, osteoporosis is expected to increase notably. Nutritional status is a relatively easily-modified risk factor, associated with many chronic diseases, and is involved in obesity, diabetes, and coronary heart disease (CHD), along with osteoporosis. Nutrients, such as fats, sugars, and proteins, play a primary function in bone metabolism and maintaining bone health. In Western nations, diets are generally high in saturated fats, however, currently, the nutritional patterns dominating in China continue to be high in carbohydrates from starch, cereals, and sugars. Moreover, high fat or high sugar (fructose, glucose, or sucrose) impart a significant impact on bone structural integrity. Due to diet being modifiable, demonstrating the effects of nutrition on bone health can provide an approach for osteoporosis prevention. Most researchers have reported that a high-fat diet consumption is associated with bone mineral density (BMD) and, as bone strength diminishes, adverse microstructure changes occur in the cancellous bone compartment, which is involved with lipid metabolism modulation disorder and the alteration of the bone marrow environment, along with an increased inflammatory environment. Some studies, however, demonstrated that a high-fat diet contributes to achieving peak bone mass, along with microstructure, at a younger age. Contrary to these results, others have shown that a high-fructose diet consumption leads to stronger bones with a superior microarchitecture than those with the intake of a high-glucose diet and, at the same time, research indicated that a high-fat diet usually deteriorates cancellous bone parameters, and that the incorporation of fructose into a high-fat diet did not aggravate bone mass loss. High-fat/high-sucrose diets have shown both beneficial and detrimental influences on bone metabolism. Combined, these studies showed that nutrition exerts different effects on bone health. Thus, a better understanding of the regulation between dietary nutrition and bone health might provide a basis for the development of strategies to improve bone health by modifying nutritional components.

## 1. Introduction

The relationship between fat, sugar, and the skeleton involves dietary nutrition composition and organ metabolism. This manuscript is organized as follows: The first section is a review of the effects of fat, fructose, glucose, and sucrose on bone metabolism through animal studies. Section 2 is a review of human-population studies related to Western dietary pattern and Chinese traditional dietary pattern effects on bone health, which also reviews the effects of bone marrow tissue, obesity, and type 2 diabetes mellitus (T2DM) on bone health.

## 2. Studies in Animals

### 2.1. High Fat Diet and Bone Health

#### High-Fat Diet and Bone Volume, Bone Microarchitecture, and Bone Strength

Five-week-old C57BL/6J mice, fed a high-fat diet (HFD) (60% kcal), had lower bone volume than those fed a low-fat diet (LFD) (10% kcal) for 12 weeks [1]. Beier et al. [1] found that, after six weeks of feeding, the bone formation marker *N*-terminal propeptide of type I procollagen (PINP) was decreased, while the bone resorption indicator tartrate-resistant acid phosphatase 5b (TRAP5b) was elevated in the HFD groups. The analyzed histomorphometrics of bone in the proximal tibia presented a significant decline in trabecular bone volume, along with an increase in bone marrow adiposity compared with those of the LFD groups. Additionally, the HFD resulted in bone marrow environment changes, might be partly responsible for bone loss [1]. Meanwhile, the osteoclast parameters (Oc.S) were significantly elevated in the HFD mice. Moreover, at the end of the experiment, the mice in the HFD group were associated with decreased stiffness and maximum force, indicating an effect on the flexural strength of femurs, which suggested that HFD not only affected bone quantity, but also bone quality [1]. Macri et al. [2] found that rats that consumed a high-saturated fatty acid diet (beef tallow) for eight weeks had the lowest total skeleton bone mineral content (BMC) and cancellous bone mineral density (spine BMD) compared with rats that consumed other types of vegetable oils. The bone mineral changes in rats might be involved in intestinal soap formation with calcium, which impaired absorption. The rats fed high-fat vegetable oil diets [2], regardless of the different omega-6(*n*-6) polyunsaturated fatty acid (*n*-6 PUFA)/omega-3(*n*-3) PUFA ratio, obtained similar total skeleton BMD, BMC, and BMC related to body weight (BMC/W) in comparison with those fed a control diet, in accordance with the American Institute of Nutrition (AIN) committee in 1993 to accommodate the increased nutritional demands of rat or mouse growth (AIN 93G) formulation. Histologic sections of tibiae presented that the rats consuming the high-saturated animal fat diet had a lower percentage of total bone volume (BV%), along with higher total alkaline phosphatase (t-AP) and bone alkaline phosphatase (b-AP), these results indicating that the AP could not be fully absorbed by subendochondral ossification, along with mineralization [2]. Six-week-old male C57BL/6J mice were given an HFD for 12, 16, or 20 weeks, respectively. Scheller et al. observed that HFD-fed mice had a significant decrease in trabecular bone volume fraction (BV/TV) and BMC, along with trabecular number (Tb.N), but that the trabecular spacing was increased relative to normal chow-diet (ND)-fed mice after 12 weeks [3]. The abovementioned bone-related parameter changes were maintained at the 16- and 20-week time points, and no further increases were observed from 16 to 20 weeks of age [3]. Cortical thickness experienced a slight reduction in the tibias of the 20-week HFD group in comparison with the ND group. In the femurs, the cortical thickness as well as the cortical tissue mineral density declined significantly in the HFD-fed mice relative to the ND-fed mice [3]. Four-point bending also demonstrated that the fracture resistance was reduced in mice fed an HFD for 20 weeks; all of these data suggest that HFD accompanied bone quality alteration, and that the marrow adipose tissue (MAT) expansion might be a factor which contributed to bone deterioration [3]. Five-week-old male Wistar rats were given a lipid-enriched diet for 10 weeks [4], and the results showed that the HFD-fed rats had a lower BMC and BMD relative to the standard diet (SD)-fed rats, which indicated that an HFD intake exerted a detrimental impact on skeletal parameters in rats during the growing period. Fat was closely related to bone metabolism and, in particular, visceral fat mass was inversely correlated with BMD.

Eight-month-old female C57BL/6J mice were fed one of three types of diet: an HFD containing monounsaturated fatty acids (MUFAs), or saturated fatty acids (SFAs), or a normal fat diet (NFD) [5]. After eight weeks of feeding, total BMC and BMD decreased in the SFA group compared to those in the NFD group [5]. The SFA group experienced increased inflammatory cytokine expression and NF-kB ligand receptor activator, which promoted bone resorption and also affected osteoblastogenesis, which eventually led to negative effects on bone. The trabecular BV/TV (%) and trabecular thickness (Tb.Th) were greater in the MUFA group compared to those in the NFD group; moreover, there was a lower structure model index (SMI) in the MUFA group relative to the other diet groups, which suggested that the MUFA diet had a beneficial effect on trabecular bone microstructure [5]. A study on ovariectomized (OVX) mice that consumed an HFD for 11 weeks showed that this sample exhibited higher levels of serum TRAP5b than those fed normal-fat diets; however, the bone formation marker osteocalcin was not affected in these mice [6]. After 11 weeks of HFD feeding, the OVX mice had a significant decrease in BV/TV and a 35% decrease in connectivity density (Conn.D). Other parameters were not significantly affected compared to the NFD group. The OVX mice fed an HFD gained more body weight, but mechanical loading did not improve the bone microstructure, which implied that the body weight increase was unfavorable to bone metabolism [6].

Czernik et al. [7] demonstrated the impact of an HFD on bone metabolism. Compared with the bone morphometric parameters of trabecular and cortical bones in 12-week-old mice fed an HFD for 11 weeks, this study found that, after 11 weeks of feeding, the bone mass was higher in the HFD group than that in the regular diet (RD)-fed group. Trabecular bone BV/TV and Tb.Th were greater in the tibia for the HFD-fed mice in comparison with those of the RD-fed mice, and were associated with a decrease in trabecular separation (Tb.Sp), along with an increased tendency in Tb.N. Moreover, cortical bone mass was increased, exclusively, in the HFD-fed mice. Dynamic histomorphometry results [7] showed that the mineral apposition rate (MAR) and bone formation rate (BFR) decreased for diet-induced obesity (DIO) animals in comparison with RD-fed mice. Bone turnover measurements also showed that the bone alkaline phosphatase (BALP) levels decreased and the TRAP5b levels increased for DIO animals, compared to those in RD-fed mice [7]. These studies were in contrast to the abovementioned work, as the DIO mice had a peak value of trabecular bone, which indicated an apparent “beneficial” impact of the HFD on bone; however, after longer feeding, the DIO mice were inclined to exhibit a proinflammatory environment, and the fat metabolism attenuated further which eventually resulted in a reduction in bone quality. In sum, the DIO animals had higher bone mass, possibly due to the initial bone formation under increasing mechanical loading and specific nutrition, along with bone anabolic adipokines, but these effects were followed by bone formation declining in response to long-term exposure to an HFD, which contributed to a decrease in bone quality and might have increased the likelihood of fractures [7].

Serum tartrate-resistant acid phosphatase (TRAP) activity increased by about 30% in the high-fat/sucrose (HFS) diet group relative to the low-fat/complex carbohydrate (LFCC) diet group [8]. Lorincz et al. [8], using 9-week-old female C57BL/6 mice consuming a HFS diet, assessed the effects of the HFS diet on bone morphology. The data showed that, after 10 weeks of feeding, there was a significant impairment in the tibia cortical bone morphology for the HFS group. The cortical thickness (Ct.Th) in the HFS diet group decreased by approximately 28% compared to the LFCC group; there was also a 25% decline in the mid-shaft cross-sectional area. Moreover, the relative load at maximum was reduced by 23% in the HFS group, which was in parallel to the LFCC group. The HFS diet-attenuated bone accretion was not only due to the enhanced bone resorption activity, but also involved in the obesity inflammatory state.

Jatkar et al., using an HFD in BALB/cByJ mice for a 15-week feeding period, found that HFD feeding markedly exerted effects on cortical and trabecular bone. That is, the cortical BV/TV (−5%), Ct.Th (−8%) and tissue mineral density (−2%) were obviously altered when comparing the HFD group and the standard chow diet group, and the trabecular bone structure was characterized by a smaller trabecular bone volume fraction (−18%) for the HFD group compared with mice fed a standard chow diet. HFD consumption had a detrimental effect on bone mass and bone microarchitecture [9]. Shu et al. [10], using 5-week-old male mice exposed to an HFD for 3, 6, and 12 weeks, examined bone volume and bone morphology. They found that the HFD-fed mice had lower trabecular BV/TV, Tb.N, and Tb.Th, and greater Tb.Sp; however, alterations to cortical bone were not significant. Histomorphometric analyses suggested [10] that the HFD-fed mice had the lower trabecular volume, but increased osteoclast numbers in the femoral metaphyseal sections after 3, 6, and 12 weeks of feeding. These findings suggested that the HFD, causing a bone mass decrease, was mainly related to the stimulation of osteoclast differentiation and activity by changing the bone marrow microenvironment.

Four-week-old C57BL/6J mice given an HFD, containing either triacylglycerol (TAG) or diacylglycerol (DAG), for 20 weeks had a higher bone mineral density (BMD) than those consuming a normal chow diet; those consuming the HFD-DAG had a higher BMD than those consuming the HFD-TAG [11]. Microcomputed tomography (micro-CT) analyses [11], conducted on the femur and lumbar vertebra, found that the cortical thickness, along with the cross-sectional area of femoral bones, increased and that there was an increase in Tb.Th of the lumbar vertebra for the HFD-DAG-fed mice, as was the case with mice fed the HFD-TAG. These findings suggested that the HFD-DAG possessed a positive action on bone metabolism and bone health compared to the HFD-TAG, which might be due to DAG promoting bone marrow cell differentiation into osteogenic cells, along with less lipotoxicity and glucotoxicity [11].

In 8 to 10-week-old male Wistar rats consuming an HFD for nine months, researchers measured bone parameters, for example, BMD, BMC, and other bone parameters, in the rats and found that there was a significant elevation in BMD, BMC, and serum t-AP in the HFD-fed rats compared to the control, normal diet-fed rats. The serum TRAP5b levels were not different between these two groups [12]. The data indicated that the bone formation increase in HFD-fed rats might be due to osteoblast activity, but did not affect osteoclast activity [12]. After shifting the diets from high-fat to normal, they found that bone markers, such as BMD, BMC, and serum TRAP5b, did not change in the HFD-fed rats [12]. Trabecular microstructure data displayed that the BV/TV and the Tb.N tend to increase, however, the Tb.Sp tends to decrease in the HFD group. Moreover, the HFD group had a lower trabecular pattern factor (Tb.Pf). A decrease in Tb.Pf means that the connected trabecular lattices are good, however, an increase indicates that the trabecular structure is more disconnected [12]. All of this suggests that HFD increased BMD, and that BMC was linked with obtaining a superior trabecular microstructure at an early stage. After ceasing the HFD, bone microstructure parameters, such as BV/TV, Tb.N, and Tb.Sp along with Tb.Pf, did not change in the HFD group, suggesting that the beneficial bone profile remained, even after ceasing the HFD [12]. 

Doucette et al., using weaned C57BL/6J male mice fed a 60% HFD for 12 weeks [13], found that areal bone mineral density (aBMD), bone length, and femoral trabecular bone BV/TV were not obviously altered, yet a slight, but marked, decrease in Tb.N and a slight increase in Tb.Sp and trabecular Conn.D occurred between the HFD-fed C57BL/6J mice and the control diet fed mice. After 12 weeks of feeding, histomorphometric measurements showed that the dynamic parameters, for instance, BFR and MAR, did not change significantly for the HFD-fed mice in comparison to regular chow diet-fed mice [13]. Moreover, the number of osteoblasts and osteoclasts did not change for both mice in the HFD-fed group, nor in regular chow-fed mice; the research on bone changes were different than previously-presented studies, in that the C57BL/6J mice fed an HFD were accompanied by significant bone loss. Both the positive and negative effects of fat diet on bone metabolism are listed in Table 1.

All of these data show that skeletal responses to obesity or HFD had either positive effects on bone, or negative effects on bone, indicating that the effects of dietary fat on bone metabolism are complex and rely on multiple factors, such as diet components, strain, feeding time, microbiome of the laboratory animals, gender, age, and mineral metabolism, along with the region of bone, and that these factors are likely to play a key role in bone metabolism. The impact of dietary fat on bone metabolism and bone microstructure involves these various factors that might exert different regulations and mechanisms and eventually affect the bone health. HFD-induced body weight and fat mass increase had both positive and negative effects on bone. On the one hand, body weight and fat mass exert mechanical loading, which promotes bone formation. On the other hand, adipocytes might impose lipotoxic effects on osteoblasts. Moreover, the HFD resulted in bone marrow adipose (BMA) expansion and the alteration of the bone marrow microenvironment, along with the proinflammatory environment, which could contribute to a negative effect on bone metabolism. Currently, how fat affects bone still is unclear and needs further study to elucidate the mechanisms by which it acts. Studies on the impact of fat on bone metabolism also provide clues as to whether changes in the lipid composition of diet could be a therapeutic strategy in preventing bone loss and in managing osteoporosis.

### 2.2. High Sugar Diet and Bone Health

#### 2.2.1. Fructose Diet and Bone Volume, Bone Microarchitecture, and Bone Strength

Bass et al. observed the influence of dietary fructose and glucose on bone formation, microarchitecture, and bone strength in an animal model [14]. The data showed that fructose diet-fed rats had higher BV/TV and lower bone surface (BS/BV) compared to those of glucose-fed rats in the region of the trabecular distal femur. Moreover, Tb.Th was greater in rats consuming the fructose diet, and a three-point bending test also showed that the maximum flexure load was obviously greater in fructose diet-consuming rats. These data implied that the rats consuming a high fructose diet for 12 weeks had a higher bone mass with a superior microarchitecture compared to those consuming a high glucose diet [14]. This study did not demonstrate the related mechanisms that introduce these changes, and the lack of a standard diet control group was a major limitation of that study. Meanwhile, Yarrow et al. [15] showed that high fructose diets had a negative effect on the skeleton of 8-week-old male Sprague-Dawley rats, and, with the incorporation of fructose diet into the HFD, the complex diets did not aggravate bone loss. Currently, the mechanisms underlying the study are not clear, and need further research. Other authors demonstrated that, after an 8-week treatment with sugar-sweetened beverages [16], 5-week-old female Sprague-Dawley rats were accompanied by increasing BMC of the whole tibia, along with increasing BMD of the proximal and distal femur; meanwhile, the calcium (Ca) intake and fecal Ca output increased, and urinary Ca excretion was reduced, which led to an increase in Ca retention in rats drinking fructose-sweetened beverages compared with those drinking glucose-sweetened beverages.

Unlike these studies, other researchers, using a fructose-rich diet (FRD), constructed the metabolic syndrome (MS) animal model and assessed the effects of FRD-induced MS on long bone histomorphometry and bone tissue regeneration [17], finding a 20% decrease in the osteocytic density of femoral trabecular bone and a 30% reduction in osteoclast-covered (TRAP-positive) bone surface in the primary spongiosa in the FRD group. However, the femoral length, trabecular bone area, and growth cartilage height did not significantly change. On the other hand, Felice et al. also evaluated the impact of FRD-induced MS on bone tissue regeneration, and found that fructose treatment simultaneously decreased reossification, significantly reduced osteocytic density, and diminished osteoclastic activity in the lesion site, which implied a decrease in bone formation and remodeling. Moreover, the study showed [17] that the FRD-induced MS decreased the ex vivo osteogenic potential of marrow stromal cells (MSC) and increased the ex vivo adipogenic potential of MSC, which was related to a reduction in Runt-related transcription factor (Runx2) and an increase in Peroxisome Proliferator Activator Receptor γ (PPARγ) expression under basal (undifferentiated) conditions. All of these data suggest [17] that the FRD-induced MS resulted in the deleterious alterations in bone microarchitecture, and in the reossification of bone lesions, and that these changes might be involved in the differentiation of adipogenic/osteogenic commitment of MSC by modulating the ratio of Runx2/PPARγ. Elevated fructose consumption in BALB/cByJ mice for 15 weeks did not significantly affect bone mass and its architecture, as well as tissue density over controls, and it was speculated that the larger brown adipose tissue mass in fructose-supplemented mice possessed an osteoprotective action [9]. When compared to the HFD-fed mice, the fructose diet-fed mice showed reduced internal stiffness, which might be associated with fructose, as well as modulated absorption of phosphorus and magnesium [18]. Clinical evidence has illustrated a link between carbohydrate consumption and whole-body macro-mineral homeostasis [19]. The data show that bone calcification was different between the fructose diet- and the HFD-fed mice groups, and all this evidence might account for the difference in the elastic modulus between the fructose diet-fed mice and the HFD-fed mice. The positive and negative effects of the fructose diet on bone metabolism are listed in Table 2.

#### 2.2.2. Glucose Diet and Bone Volume, Bone Microarchitecture, and Bone Strength

For rats provided sugar-sweetened beverages, BMD in the femur was decreased for rats consuming glucose-sweetened beverages in comparison with rats consuming sucrose- and fructose-sweetened beverages [16]. Using peak force, bending failure energy, and ultimate stiffness, along with ultimate bending stress to evaluate bone strength, no significant differences were found among these groups. The Ca and P intakes and fecal Ca output were lower; however, Ca absorption was higher in the rats fed glucose-sweetened beverages than the rats fed fructose, along with high-fructose corn syrup (HFCS-55)-sweetened beverages. Another possible mechanism for femur and tibia BMD reduction might be due to glucose exerting a direct effect on bone. Some studies have shown that [25] high concentrations of glucose suppressed the differentiation and proliferation of osteoblasts, which, in turn, can attenuate bone formation and, eventually, bone mass loss. In accordance with bone health, an increase in glucose diet consumption could be of concern to researchers, rather than the current focus on fructose diet consumption elevation associated with HFCS-55 beverage intakes [16]. Both the positive and negative effects of glucose diet on bone metabolism are listed in Table 2.

#### 2.2.3. Sucrose Diet and Bone Volume, Bone Microarchitecture, and Bone Strength

Gerbaix et al. [20] observed that a normocaloric high-fat/high-sucrose (HF/HS) diet improved whole-body BMD and bone mass, while trabecular bone volume in the tibia and vertebrae persisted. Growing and mature rats ingested an isocaloric HFS diet for 19 and 27 weeks to induce obesity-related bone metabolism disorder [21], and it was observed that the HFS diet seemed to favor trabecular bone mass, while cortical thickness, along with density, did not obviously change.

Contrary to these studies, Weaning Wistar male and female rats received a high sucrose diet for 5 weeks [22], and it was observed that both the male and female rats had significantly lower bone strengths than those of the control diet group. For the female rats, the femur and tibia weight, the tibia width, and the Ca and P concentrations in bone were markedly lower in the sucrose-fed group than those in the control diet group. These data suggest that the sucrose diet, which induced metabolic disturbance, might explain the differences in bone metabolism.

Smith et al. [26] examined the influence of the composition of age (3, 8, 16, and 56 weeks) and diet (high sucrose, high fat) on Sprague-Dawley rat bone mechanical properties, and they found that, after 5 weeks of feeding, the size, composition, and mechanical properties of bone for Sprague-Dawley rats were not affected by being fed a high sucrose diet. Whether a longer feeding time or different diet composition would result in changes was not clear and needs further study. Lorincz et al. [8] performed a study on 9-week-old female C57BL/6 mice given an HFS diet for 10 weeks, which exerted a detrimental impact on bone structure and morphological properties in cortical bone. These effects were likely involved in increased osteoclast activity, which was linked with the inflammatory environment. Long-term (24 months) ingestion of an HFS diet for female rats, also had an adverse effect on BMC, histology of the femoral neck and vertebrae, along with mechanical properties [23]. Similarly, Salem et al. study [24] suggested that 8-week-old female Sprague-Dawley rats fed an HFS diet for 10–12 weeks experienced a negative impact on vertebrae mechanical properties.

In summary, the abovementioned studies imply that saccharide diet consumption might exert different impacts on bone mass, morphology, microarchitecture, and mineralization; excess sugar intake resulted in metabolism disturbance, and long-term high-sugar diet consumption might increase the risk of developing metabolism disorders in the future. Thus, we should control dietary sugar intake, which might provide an opportunity to improve individual bone health. Both the positive and negative effects of sucrose diet on bone metabolism are listed in Table 2.

## 3. The Effect of Fat and Sugar Diet on Bone Health among Human Population Studies

Bone is a dynamic organ that has a complex internal structure. Regarding the physiological conditions, bone formation and resorption maintain a dynamic balance. If the number of osteoclasts increases and the number of osteoblasts are inadequate, this will lead to bone loss, and, eventually, the occurrence of osteoporosis. Globally, osteoporosis has increased rapidly. Multifaceted determinants of osteoporosis include metabolism disorders, genetic background, lifestyle, nutrition, and medication use. Evidence has demonstrated the importance of nutrient compositions in retaining BMD and defending against osteoporosis and osteoporotic fractures.

### 3.1. Different Dietary Patterns and Bone Health

Previous studies have illustrated that MS correlates with a higher risk of bone loss and bone fragility. Another study also reported that hip fracture incidences have declined in Asians, relative to several non-Asian populations [27,28].

A cross-sectional study compared the Chinese traditional dietary pattern and the Western-style diet with respect to bone metabolism in Chinese freshmen [29], and the findings indicated that the Chinese traditional dietary pattern was negatively related to the risk of osteopenia/osteoporosis and maintained a healthy body mass index (BMI), while the Western diet increased the risk of overweight/obesity. Moreover, to investigate the disparities of dietary structures, as well as to analyze the impacts of different dietary patterns on bone mineral status among students from urban and rural China, a study by Yang et al. showed that students from urban areas consumed more Chinese, Westernized, and meat diets than students from rural areas. However, rural students had a lower BMD, and the Chinese and Western dietary patterns could decrease the low bone mineral quality risk, which was also presented in this study [30]. Zeng et al. [31] studied the relationship of the four different dietary patterns; healthy diets, prudent diets, traditional diets, and high-fat diets, relative to hip fracture risk in elderly Chinese populations in a coastal region of China, and the results demonstrated that the healthy dietary pattern, characterized by a high fruit and vegetable intake, was related to a lower hip fracture risk. A high intake of nuts, mushrooms, algae, and seafood, but low intake of grains, characterized the prudent dietary pattern, which was also related to a lower hip fracture risk. In contrast, the high-fat dietary pattern was related to a higher hip fracture risk. In addition, the traditional dietary pattern was not found to be significantly correlated with hip fracture risk [31]. On the other hand, based on the study by Wang et al. [32], Chinese people living in Denmark in excess of 12 years had a BMD that resembled that of the Danish population; however, people living there fewer than 12 years had a lower BMD. This is likely linked with a more Westernized lifestyle and dietary pattern.

Comparing the Chinese traditional dietary pattern and the Western dietary pattern, these studies suggested that we should follow the traditional Chinese diet. Since China is the largest developing country, having experienced significant economic development over the last three decades, the Chinese people have undergone a nutritional transition and have established a more Western-style diet; the Western dietary pattern is usually accompanied with overweight/obesity and increased central obesity risk. Based on this, we suggest that that we should follow the traditional Chinese diet and restrict the Western diet, which would help maintain good bone health.

The data from the North West Adelaide Health Study, which is a study in Australia, found the that the Western pattern diet was accompanied by a prevalence of low BMD, but the BMD levels were increased in people with a prudent pattern diet [33]. To study the relationship between dietary patterns and hip fractures, 74,540 postmenopausal women and 35,451 men above the age of 50 were followed from 1980–2010 and 1986–2012, respectively [34]. During the follow-up, a total of 1891 women and 596 men had suffered hip fractures, and the results did not find a relationship between the Western pattern diet and hip fracture risk in women or men. In this study, the analysis was confined to a Caucasian population, and additional ethnic populations need to be investigated [34]. Langsetmo et al. performed a retrospective cohort study on the basis of the Canadian Multi-Center Osteoporosis Study, and investigated the association of dietary patterns and fracture incidences, observing that energy-foods, characterized by a high level of soft drinks, potato chips, French fries, meats, and desserts, were not associated with fractures; however, nutrient-dense foods, such as fruit, vegetables, and whole grains, were correlated with a lower fracture risk. Due to older women being more inclined to fracture risk, these findings encourage an increase in nutritious food intake, which is beneficial to decreasing fracture risk [35]. For postmenopausal women who were enrolled in the Korean Genome and Epidemiology Study, the results implied that the Western dietary pattern demonstrated a higher risk for osteoporosis in postmenopausal Korean women [36]. Okubo et al. [37] analyzed the effects of dietary patterns on BMD among 291 premenopausal Japanese farmwomen, and the study indicated that the Western dietary patterns had a trend that was inversely associated with BMD. One cross-sectional study investigated 3236 post-menopausal Scottish women [38], and a poor dietary pattern (processed foods and snack foods) was related to a decreased BMD. Similarly, an energy-dense, nutrient-poor dietary pattern is correlated with decreased total body BMC [39]. The aim of another study was to discuss the impact of diet patterns on bone metabolism in a cohort of Canadian adults [40], and the Western diet pattern was related to an increase in bone turnover (high bone-specific alkaline phosphatase and lower 25-OHD in women; higher *C*-terminal telopeptide (CTX) in men), which might result in increased fracture risk [40]. With respect to the relationship between dietary patterns and BMD, a study that investigated Brazilian postmenopausal women found that sweet foods and caffeinated beverages were negatively related to BMD, and no association was found between the Western dietary pattern and BMD [41].

Based on these studies, we found that the Western diet (energy-dense) pattern is usually inversely associated with BMD in both men and women, particularly in postmenopausal women. It is well known that hip fractures are a main cause of morbidity in the elderly, and fractures also result in decreased quality of life, disability, and increased mortality. Considering the long-term effects of low BMD, treatment of subjects who had a lower BMD might benefit from a decreased fracture risk. Thus, for these subjects, the intake of nutrient-dense foods, rather energy-dense foods, might be a good diet strategy for maintaining bone health. The effects of different dietary patterns on bone health are listed in Table 3.

### 3.2. Bone Marrow Tissue and Bone Health

The association of between fat and bone is complex, and is not only involved in systemic effects, but is also related to local effects. Local action mainly refers to the activity of fat on the bone marrow microenvironment, along with interactions with other bone cells [42]. Bone marrow adipose tissue (BMAT) serves as a distinct fat depot, and the vital role that it plays in fat–bone interactions has been proved [43]. In vitro and animal studies have provided a great deal of data focused on the association of bone marrow fat and bone, and the literature has been widely reviewed [42,43,44,45,46,47]. In human studies, an inverse relationship has been shown between bone marrow fat and BMD, in both healthy subjects and ostoepenic/osteoporotic patients. On the other hand, other metabolic diseases, such as anorexia nervosa (AN), T2DM, and obesity, also affect the bone marrow fat [48]. The bone marrow fat at the fourth lumbar vertebra and femur increased for AN subjects, which was negatively correlated with the BMD of the spine and hip [49]. Baum et al. [50] compared the bone marrow fat content of vertebral and lumbar spine volumetric bone mineral density (vBMD) in postmenopausal women, with and without T2DM. These findings showed that mean bone marrow fat was no different in these two groups of subjects, and that mean bone marrow fat was inversely correlated to vBMD. Moreover, diabetic subjects had significantly lower unsaturated lipid fractions. The findings of Patsch et al. [51] demonstrated that bone marrow and unsaturated lipid contents were lower, and that saturated lipid contents were higher, in diabetics with fractures; no association was observed between bone marrow fat and diabetes with respect to fracture risk. In a cross-sectional study of 35 obese (mean BMI 36.5 ± 5.8 kg/m^2^) men at a clinical research center [52], it was observed that the content of bone marrow fat in vertebrae was negatively correlated to the vBMD cortical area, along with Tb.Th. With the development of imaging techniques, the role of adiposity on skeletal health can be studied directly and noninvasively; bone marrow fat can be noninvasively quantified using magnetic resonance imaging (MRI), either with or without spectroscopy, or by using MRI with spectroscopy (MRS) in combination with dual-energy X-ray absorptiometry (DXA) [48]. Additionally, it has been reported that the bone marrow fat of vertebrae can be determined using proton magnetic resonance spectroscopy (1H-MRS) [50]. Measurement and understanding of the relationship between BMT and bone has recently become a hot research topic. In clinical practice, the usefulness of only evaluating BMT using MRI (for clinicians) is still limited, and is critical to further understand its functional actions and implications for bone remodeling. All of these imaging techniques will benefit the diagnosis and management of bone metabolism-related diseases.

### 3.3. Obesity and Bone Health

Obesity and osteoporosis have become global health problems, resulting in worldwide high mortality and morbidity risk. The traditional viewpoint was that obesity was advantageous to bone health due to the positive action of mechanical load, caused by body weight, on bone formation, and that it helped to protect against osteoporosis [53,54]. Recent studies, however, have shown that obesity imposes a negative effect on bone metabolism, leading to osteoporosis [55,56,57,58].

Adipose tissue is usually considered as a passive energy reservoir; in fact, adipose tissue is an active endocrine organ that secretes cytokines, named adipokines, such as leptin and adiponectin. Greco et al. [59] have pointed out that adipose tissue, which can modulate skeletal metabolism, might depend on adipokines to regulate bone remodeling via their action on either bone formation or resorption. Moreover, adipose tissue also secretes proinflammatory cytokines, such as interleukin 6 (IL-6) and tumor necrosis factor alpha (TNF-α), which might promote osteoclast differentiation, resulting in enhanced bone resorption [60]. The abovementioned molecules differentially modulate bone metabolism, which leads to the complex relationship between adipose tissue and bone [56,61]. The mechanisms underlying these events are not clear and require further study.

### 3.4. T2DM and Bone Health

Fragility fractures, considered to be an important complication of diabetes, have been recognized [62], and are related to substantial morbidity and mortality, and impose a tremendous economic burden on healthcare [62,63]. T2DM patients usually have normal or high BMD [64,65]. In fact, bone strength, stiffness, failure load, and cortical load fraction were shown to significantly decrease in patients with T2DM [65,66]. Bone fragility results from bone mass loss and also from the impairment of bone microstructure. MRIs have shown that T2DM patients tend to have greater cortical porosity [67]. Similarly, high-resolution, peripheral, and quantitative CT (HRpQCT) has implied a trend, or increase, in cortical porosity in postmenopausal women with T2DM [65,66,68]. All of these studies suggest that bone quality decreases among T2DM subjects. The pathophysiological mechanisms underlying bone fragility in T2DM are complex, and hyperglycemia is a primary factor that inversely modulates the function of osteoblasts and bone formation; diabetic mellitus (DM) induces lipid accumulation in the bone marrow, which is susceptible to a differentiated decrease in osteoblasts [69]. Meanwhile, advanced glycation end products (AGE) or non-enzymatic cross-links within collagen fibers are responsible for bone structure and impair mechanical properties [70]. Additionally, dysautonomia and impaired leptin function might result in osteopenia/osteoporosis in DM [69]. When β-cell activity decreases, and was successively accompanied by worsening glucose control, bone mineralization and strength also deteriorated, resulting in bone health impairment in DM patients. Medications with a neutral or favorable action on bone metabolism, such as metformin- and incretin-based therapies, have been advised to be preferentially applied; however, no relevant guidelines indicate how or at which stage of the disease anti-osteoporotic treatment should be applied in DM patients.

Due to modern industrial developments, the availability of sweeteners has increased, and sweeteners are often added to diets via sugar-sweetened beverages, such as soft drinks and fruit-flavored drinks [71]. The association of soft drink consumption and low BMD, along with fractures, has been reported in pubescent girls [72]. In addition, research conducted on humans has illustrated the association of fructose and glucose intake with poor bone health [19,73].

In human studies, most reports have demonstrated that a high-fat, high-sugar diet exerts adverse effects on bone mass, and is also associated with fracture development. In clinical practice, it is more beneficial to provide suggestions on dietary patterns, rather than medicine, to provide a protective effect on bone health.

## 4. Conclusions

Fat and sugar diets exert different effects on bone metabolism. Due to dietary fat and sugar intakes dramatically increasing over the last century, numerous studies have demonstrated the impacts of dietary fat and sugar on bone metabolism, and have confirmed that dietary nutrition possesses complicated effects on bone health. However, the current mechanisms for the relationship between dietary intake and bone health are not clear, and further study is needed to elucidate these mechanisms. Most studies have demonstrated that dietary interventions might be a means by which benefits to bone development and fracture prevention may occur. We should pay more attention to the relationship between dietary nutrition composition and bone metabolism.

## Figures and Tables

**Table 1:** Effect of fat diet on bone metabolism.

Strain	Age (Weeks)	Gender	Fat Amount in Diet	Fat Types in Diet	Feeding Time (Weeks)	Effect on Bone	References
**Negative**							
C57BL/6J mice	5	Male	60% kcal	Lard/Soybean Oil	12	Femur trabecular. BV/TV↓; Tb.N↓; Conn.D↓; Tb.Sp↑; stiffness↓; max force↓; P1NP↓; TRAP5b↑	[1]
Wistar rat	3	Male	40% kcal	Beef Tallow	8	Total skeleton BMC↓; BMC/W↓; spine BMD↓; the BV%↓; t-AP↑; b-AP↑	[2]
C57BL/6J mice	6	Male	60% kcal	Lard/Soybean Oil	12, 16, 20	Femur and tibia BVF↓; BMC↓; Tb.N↓; Tb.Sp↑; femur yield load↓; post-yield ↓	[3]
Wistar Rat	5	Male	38.5% kcal	Vegetable Oil	10	BMC↓; BMD↓; SA↓	[4]
C57BL/6J mice	32	Female	44% kcal	Lard/Soybean Oil/Coconut Oil (SFA)	8	Total body and femur BMD↓; total body BMC↓; cortical BMD↓, cortical porosity↑	[5]
C57BL/6 mice	16	Ovariectomized Female	45% kcal	Lard/Soybean Oil	11	Proximal tibia trabecular BV/TV↓; Tb.N↓; Tb.Th↓; Conn.D↓; SMI↑; Tb.Sp; TRAP↑	[6]
C57BL/6 mice	12	Male	45% kcal	Lard/Soybean Oil	11	Trabecular total BMD↑; tibia trabecular BV/TV↑; Tb.Th↑; Tb,N↑;Tb.Sp↓; PMoI↑; Imax/Cmax↑; MAR↓; BFR↓; BALP↓;TRAP5b↑	[7]
C57BL/6 mice	9	Female	39.5% kcal	Lard/Maize Oil	10	Tibia mass↓; tibia length↓; Ct.Th↓; cross-sectional area↓; maximal load↓; flexural rigidity↓; TRAP↑	[8]
BALB/cByJ mice	7	Male	45% kcal	Lard/Soybean Oil	15	Distal femur cortical BV/TV↓; Ct.Th↓; tissue mineral density↓; trabecular BV/TV↓; Tb.N↓;SMI↓; Tb.Sp↑	[9]
C57BL/6J mice	5	Male	60% kcal	Lard/Soybean Oil	3, 6, 12	Femoral trabecular BV/TV↓; Tb.N↓; Tb.Th↓; Tb.Sp↑	[10]
**Positive**							
C57BL/6J mice	4	Male	45% kcal	Lard/DAG; Lard/Soybean Oil;	20	BMD↑; femoral cortical thickness↑; cross-sectional area↑; Tb.Th in vertebrae↑	[11]
Wistar rat	8–10	Male	24% kcal	Groundnut/coconut	36	BMD↑; BMC↑; Tibia trabecular BV/TV↑; Tb.N↑; Tb.Sp↓; Tb.Pf↓; T-ALP↑	[12]
C57BL/6J mice	32	Female	46% kcal	Lard/Soybean Oil/Olive Oil (MUFA)	8	Femur trabecular BV/TV↑; Tb.Th↑; SMI↓	[5]
**Neutral**							
7BL/6J mice	3	Male	60% kcal	Lard/Soybean Oil	12	Femur trabecular BV/TV; Tb.Th; Tb.Sp; cortical bone parameters; BFR MAR not different	[13]

BV/TV, bone volume fraction; Tb.N, trabecular number; Tb.Th, trabecular thickness; Conn.D, trabecular connectivity density; Tb.Sp, trabecular separation, SMI, trabecular structural model index, P1NP, N-terminal propeptide of type I procollagen; TRACP5b, tartrate-resistant acid phosphatase 5b; BMC, bone mineral content; BMD, bone mineral density; BMC/W, BMC was expressed per body weight; t-AP, total alkaline phosphatase; b-AP, bone alkaline phosphatase; BVF, trabecular bone volume; SA, skeleton area; Tb.Pf, trabecular pattern factor; Ct.Ar, cortical bone area; Ct.Th, cortical bone thickness; SFA, saturated fatty acids; MUFA, monounsaturated fatty acids; BFR, bone formation rate; MAR, mineral apposition rate; ↓ denotes decrease; ↑ denotes increase.

**Table 2:** Fructose, glucose, and sucrose diet effect on bone metabolism.

Strain	Age (weeks)	Gender	Feeding Time (Weeks)	Feeding Dose	Effect on Bone	References
**Positive**						
Sprague-Dawley rat	8–9	Male	12	40% Fructose	Femur trabecular BV/TV↑; Tb.Th↑; BS/BV↓; maximum flexure load↑	[14]
Sprague-Dawley rat	8	Male	12	40% Fructose	Distal femur Ct.Th↑; Ct.Ar↑; vTMD↑; femoral diaphysis Ct.Th↑; Ct.Ar↑; vTMD↑	[15]
Sprague-Dawley rat	5	Female	8	13% Fructose	Whole femur and tibia BMC↑; BMD↑ ^a^	[16]
**Negative**						
Sprague-Dawley rat	Adult	Male	4	10% Fructose	Osteocyte density↓; metaphyseal relative trabecular bone area↓; TRAP area/trabecular bone area↓; ALP activity↓; Type 1 collagen production↓	[17]
Sprague-Dawley rat	5	Female	8	13% Glucose	Femur weight↓; whole femur, tibia BMC↓ ^b^; whole femur, tibia BMD↓ ^c^	[16]
**Positive**						
Wistar rat	28	Male	16	22% Sucrose	Whole BMD↑; positive uncoupling index; osteoid surface on tibia↑; cortical porosity↓	[20]
Wistar rat	4, 24	Male	19, 27	22% Sucrose	Trabecular BV/TV↑	[21]
**Negative**						
Wistar rat	3	Male, Female	5	43% Sucrose	The weights of tibias and femurs↓; the final width of the tibias↓; densities of tibias and femurs↓; the breaking strength of the tibias and femurs↓	[22]
C57BL/6	9	Female	10	47% Sucrose	Tibial mass↓; tibial length↓; Ct.Th↓; cross-sectional area↓; maximal load↓; energy to failure↓; flexural rigidity↓	[8]
Fischer 344 rat	4	Female	96	39.5% Sucrose	Trabecular core ↑; cortical shell↓ in the femoral neck; cross-sectional area↓ in the sixth lumbar vertebra; loads↓, energies↓, stiffnesses↓ in the femoral neck and sixth lumbar vertebra	[23]
Sprague-Dawley rat	8	Female	10–12	39.5% Sucrose	Cross-sectional area↓, height↓, volume↓ in the sixth lumbar vertebra; loads↓, energy↓, stiffness↓ in the sixth lumbar vertebra	[24]

BV/TV, bone volume fraction; Tb.Th, trabecular thickness; Tb.Sp, trabecular separation, TRAP, tartrate-resistant acid phosphatase; BMC, bone mineral content; BMD, bone mineral density; ALP, alkaline phosphatase; Ct.Ar, cortical bone area; Ct.Th, cortical bone thickness; vTMD, cortical volumetric tissue mineral density. ^a^ denote compared to the glucose sweetened beverage; ^b^ denote compared to the fructose-sweetened beverages; ^C^ denote compared to all the other sugar-sweetened beverage groups.

**Table 3:** Different dietary patterns and bone health in human studies.

Study, Location	Subjects Information	Dietary Pattern	Method Measurement	Results	References
A cross-sectional study in China	1319 college freshmen from four universities (aged 18.1 ± 1.2 years)	Western food; animal protein; calcium food; Chinese traditional patterns	Ultrasound bone densitometer by measuring speed of sound	Chinese traditional dietary pattern positive correlation with BMD	[29]
A cross-sectional study in urban and rural of China	1590 students from two primary schools and two junior high schools (aged 11–17 years)	Westernization structure; meat diet structure; Western and Chinese structure	Ultrasound bone densitometer by measuring speed of sound	Chinese and Western dietary pattern was negative associated with the low bone mineral quality risk	[30]
A matched case-control study in China	581 cases including 396 cases of femoral neck fractures and 185 cases of intertrochanteric fractures; 581 eligible age, and gender-matched controls from either communities or hospitals (aged 50–80 years)	Healthy dietary pattern; prudent dietary pattern; traditional dietary pattern; high-fat dietary pattern	X-ray	High-fat dietary pattern was associated with high incidence of hip fractures; healthy and prudent dietary patterns could be associated with low incidence of such fractures	[31]
A cross-sectional study in immigrants from southern China to Denmark	73 women (aged 35 ± 8 years) and 69 men (aged 40 ± 12 years) who immigrated to Denmark from 2 months to 36 years		Dual energy X-ray absorptiometry	Chinese women who had immigrated to Denmark more than 12 years ago had similar BMD to Danish women; those who had immigrated less than 12 years ago had a lower BMD	[32]
North West Adelaide Health Study in Australia	1182 adults (545 males, 45.9%) (aged 50 years and above)	Prudent pattern; Western pattern	Prodigy and DPX+ dual energy X-ray absorptiometry	The Western pattern was associated with a higher prevalence of low BMD	[33]
A prospective cohort study and health professionals’ follow-up study in the US	1891 cases of hip fractures in women and 596 in men	Prudent pattern; Western pattern	Self-reporting of fractures and medical record review	Both the prudent and the Western dietary patterns were not associated with the risk of hip fractures in postmenopausal women or men over 50 years of age	[34]
A retrospective cohort study based on the Canadian Multicentre Osteoporosis Study	5188 enrolled in the study cohort, 3539 were women and 1649 were men	Nutrient-dense pattern; Energy-dense pattern	Hologic densitometers; GE/Lunar densitometers	The energy-dense pattern was not associated with fractures; however, the nutrient-dense pattern was negative associated with low-trauma fracture risk	[35]
A cohort and follow-up study	1464 postmenopausal Korean women	Korean traditional dietary pattern; dairy dietary pattern; Western dietary pattern	Quantitative ultrasound measurement by measuring the speed of sound	The Korean traditional dietary pattern and Western dietary pattern were associated with a high risk of osteoporosis incidence; the dairy dietary pattern decreased the risk of osteoporosis	[36]
Japanese Multicentered Environmental Toxicant Study (JMETS)-based study of farmwomen	291 premenopausal farmwomen (aged 40–55 years)	Healthy dietary pattern; Western dietary pattern	Dual energy X-ray absorptiometry	The Western dietary pattern was negatively associated with BMD, however, the healthy dietary pattern was positively associated with BMD	[37]
A cross-sectional study in a Scottish population	3236 Scottish women (aged 50–59 years)	Healthy foods; processed foods; snack foods; bread and butter; fish and chips	Dual-energy X-ray absorptiometry	Processed foods and snack foods were associated with a lower BMD; however, the healthy pattern was associated with a high BMD	[38]
A study in the Twin and Sister Bone Research Program at Royal Melbourne Hospital in Australia	527 women (aged 18–65 years)	Five dietary patterns	Dual-energy X-ray absorptiometry	The energy-dense dietary pattern was negatively associated with BMD; however, the nutrition-dense dietary pattern was positively associated with BMD	[39]
A cohort study in Canadian adults	754 women and 318 men	Prudent diet; Western diet	B-ALP; CTX; 25OHD	The Western diet was inversely associated with bone metabolism	[40]
A cross-sectional study in Brazilian women	156 postmenopausal and osteoporotic Brazilian women (aged over 45 years)	Healthy diets; red meat and refined cereals; low-fat dairy; sweet foods, coffee and tea; Western diet	Dual-energy X-ray absorptiometry	The sweet foods, coffee and tea pattern was inversely related to BMD	[41]

BMD, bone mineral density; B-ALP, bone-specific alkaline phosphatase; 25OHD, 25-hydroxyvitamin D; CTX, *C*-terminal telopeptide.
